# Radiomics in the Evaluation of Cystic and Neoplastic Lytic Lesions of the Jaws

**DOI:** 10.3390/diagnostics16081222

**Published:** 2026-04-20

**Authors:** Paola Di Giacomo, Pasquale Frisina, Alberto Fratocchi, Pierluigi Barra, Cira Rosaria Tiziana Di Gioia, Flavia Adotti, Giovanni Falisi, Fabrizio Spallaccia, Iole Vozza, Antonella Polimeni, Carlo Di Paolo, Daniela Messineo

**Affiliations:** 1 Department of Oral and Maxillofacial Sciences, Sapienza University of Rome, 00161 Roma, Italy; iole.vozza@uniroma1.it (I.V.); antonella.polimeni@uniroma1.it (A.P.); carlo.dipaolo@uniroma1.it (C.D.P.); 2 Department of Radiology, Pathology and Oncology, Sapienza University of Rome, 00161 Rome, Italy; pasquale.frisina@uniroma1.it (P.F.); cira.digioia@uniroma1.it (C.R.T.D.G.); daniela.messineo@uniroma1.it (D.M.); 3 Oral Surgery, Policlinico Umberto I Hospital, 00161 Rome, Italy; al.fratocchi@policlinicoumberto1.it; 4 Maxillofacial Surgery, Policlinico Umberto I Hospital, 00161 Rome, Italy; p.barra@policlinicoumberto1.it; 5 Department of Experimental Medicine, Sapienza University of Rome, 00161 Rome, Italy; flavia.adotti@uniroma1.it; 6 Department of Life, Health and Environmental Sciences, University of L’Aquila, 67100 L’Aquila, Italy; giovanni.falisi@univaq.it; 7 Maxillofacial Surgery, Terni Hospital, 05100 Terni, Italy; f.spallaccia@aospterni.it

**Keywords:** radiomics, lytic lesion, odontogenic cysts, jaw tumors, machine learning, cone-beam computed tomography

## Abstract

**Background/Objectives.** Radiomics is an emerging imaging-based tool that enhances lesion characterization beyond conventional diagnostic approaches. Its potential in evaluating osteolytic lesions of the jaws lies in improving discrimination between benign and malignant entities. This study aimed at developing a predictive model to identify radiomic features capable of distinguishing benign from malignant lesions. **Methods.** Subjects with preoperative CT or CBCT and histopathological confirmation were included. A pilot cohort was used for feature selection via LASSO regression, which ranked features by frequency and absolute coefficient. Malignancy was coded as class 1, benign lesions as class 0. Positive coefficients indicated association with malignancy, while negative coefficients with benign characteristics. The most stable features were initially trained on the pilot cohort and then validated on an independent test set through machine learning classifiers as LASSO, support vector machine, artificial neural network, random forest e XGboost. **Results.** The sample comprised 69 subjects (pilot cohort = 57, test cohort = 12). The predictors selected from LASSO regression were: DifferenceEntropy_GLCM (−0.768), CenterOfMassShift_MORPHOLOGICAL (−1.390), INTENSITY-HISTOGRAM_MaximumHistogramGradientGrayLevel (1.139), GLRLM_ShortRunLowGrayLevelEmphasis (−0.742), and Maximum3DDiameter_MORPHOLOGICAL (0.932). As for model performance on test, LASSO achieved the best performance (AUC 0.83), with perfect specificity and sensitivity of 0.71. SVM showed good AUC but poor sensitivity, while random forest and XGBoost performed poorly (AUC 0.57 and 0.37, respectively). **Conclusions.** The LASSO model proved to be a transparent and robust classifier, suitable for both feature selection and external validation. The selected features demonstrated strong discriminative ability, supporting the potential of radiomics in improving lesion assessment and guiding clinical decision-making.

## 1. Introduction

Lytic lesions of the jaws represent a heterogeneous and complex group of pathologies characterized by the destruction of bone tissue (osteolysis) in the maxilla and mandible. Given their diverse nature, these lesions require careful differential diagnosis, as they encompass a broad spectrum of etiologies, each associated with distinct therapeutic approaches [1].

**Figure 1:**
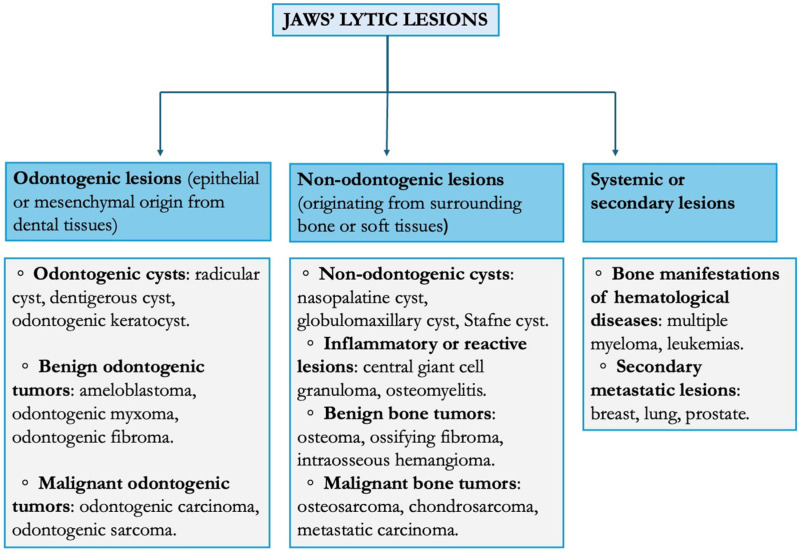
Classification of osteolytic jaw lesions.

Lytic lesions can be classified based on their origin [2,3] as shown in Figure 1.

The diagnostic framework is based on the integration of clinical, radiographic, and histological data. During the medical history and clinical examination, signs such as swelling, unexplained dental mobility, and mucosal involvement, together with symptoms such as pain or paresthesia of the inferior alveolar nerve, may be observed. Imaging plays a crucial role in formulating the diagnostic hypothesis: panoramic radiography is a first-line examination; CT (dentascan, cone-beam, or facial CT) is essential for lesion characterization; and MRI is indicated when soft-tissue involvement must be assessed. The definitive diagnosis is established by histopathological examination, which guides the therapeutic approach.

Diagnosing lytic lesions can be challenging due to radiographic overlap across categories and limitations in biopsy sampling, as well as overlapping histopathologic features that may compromise differential diagnosis [4,5]. In this context, radiomics is gaining increasing attention. In fact, accurately distinguishing benign from malignant osteolytic lesions of the jaws is a critical step in clinical practice. Although many of these lesions share overlapping radiographic features, their management differs substantially: benign entities may be treated conservatively or with limited surgical intervention, whereas malignant lesions require prompt diagnosis, immediate biopsy, and more aggressive treatment, often within a multidisciplinary oncologic setting. A reliable imaging-based assessment can therefore reduce unnecessary biopsies, shorten diagnostic delays, and improve treatment planning. In this context, advanced quantitative approaches such as radiomics offer the potential to extract subtle imaging features not appreciable to the human eye, thereby enhancing the ability to discriminate between lesions with different biological behaviors.

Radiomics enables high-throughput extraction of quantitative features from digital imaging, based on the principle that microscopic cellular and subcellular characteristics are reflected in macroscopic tissue patterns. Radiomics analysis follows defined phases [6,7]:

- Imaging protocol and delineation: Acquisition of high-quality images and segmentation of the volume of interest (VOI), performed manually, semi-automatically, or automatically.
- Feature processing and extraction: Image processing, feature extraction, and selection.
- Feature classification: Features are grouped as shape-based (geometry of the VOI, e.g., volume, 3D diameter), first-order (distribution of voxel values independent of spatial position), and textural (spatial relationships between voxel intensities).

The goal is to identify textures—pixel/voxel relationships imperceptible to the human eye—that correlate with underlying biological information and that may provide noninvasive imaging biomarkers that complement histopathology. Radiomics thus supports personalized diagnosis, prognosis, and prediction of treatment response, particularly in oncology.

Its application to lytic jaw lesions is of scientific interest, as it may help characterize lesions that are difficult to differentiate on imaging prior to histology. Benign lesions are often treated invasively to exclude malignancy, while malignant lesions may be misclassified or diagnosed late.

The aim of this study was to investigate whether quantitative radiomic features extracted from CBCT/CT images can reliably differentiate benign from malignant osteolytic lesions of the jaws. To this end, we analyzed a pilot cohort including cysts, ameloblastomas, keratocysts, and carcinomas/sarcomas of the maxilla and mandible, identifying the most clinically meaningful discriminative features. These features were then used to develop a predictive model, which was subsequently validated on an independent test cohort using a machine learning protocol. By improving the non-invasive characterization of jaw lesions, this research has the potential to support earlier and more accurate diagnostic decision-making and enhance treatment planning in routine clinical practice.

## 2. Materials and Methods

The research was conducted at the Head and Neck Department of Policlinico Umberto I, Sapienza University of Rome. All procedures involving human participants complied with the ethical standards of the 1964 Declaration of Helsinki and its subsequent amendments [8]. The study was approved by the Institutional Ethics Committee (Protocol No. 0002427/2025). All patients provided informed consent for data processing, and all imaging and clinical information were anonymized prior to analysis.

Given the relatively low incidence of the pathology under investigation, no a priori sample size calculation was planned. Given the exploratory nature of the study and the use of machine learning techniques, statistical power was not calculated using classical frequentist criteria. In radiomics, sample size evaluation must be interpreted differently from that in traditional clinical studies: the primary objective is not to test statistical hypotheses about individual variables, but rather to construct multivariate predictive models that learn complex patterns from the data.

Patients treated at Policlinico Umberto I (dental/maxillofacial clinical section) between November 2023 and October 2025 were retrospectively enrolled if they had a diagnosis of benign lytic lesion (cysts, keratocysts, ameloblastoma) or malignant lesion of the jaws (sarcoma or squamous cell carcinoma), with both a preoperative CT scan and a definitive histopathological diagnosis available in the institutional archive. The choice to include only these lesions was based on their higher frequency and the need to reduce internal variability within the sample.

### 2.1. Inclusion Criteria

1. High-quality preoperative CT imaging and a lesion (>10 mm in the largest dimension) to allow radiomic analysis.
2. Definitive histopathological confirmation of the lesion following surgical resection.

All patients meeting the inclusion criteria during the study period were enrolled consecutively.

All CT images were stored in DICOM (Digital Imaging and Communications in Medicine) format. Patients were divided into two groups: a “development cohort” (approximately 80% of the total sample) used for feature analysis, model construction, and training, which, for simplicity, we refer to as the “PILOT COHORT”, and an “INDEPENDENT TEST COHORT” (approximately 20%) used for final model validation. To ensure reproducibility, all dataset partitions were generated using a fixed random seed. A stratified splitting strategy was adopted to maintain the relative class distribution across the training, validation, and test sets.

Segmentation was performed on noncontrast CT images to analyze the lesion’s intrinsic characteristics rather than the distribution of contrast material. Manual segmentation was performed by an experienced operator (a dentist with >5 years of experience in maxillofacial imaging) using LIFEx software (version 7.6) [9]. All segmentations were subsequently checked by a second experienced operator to ensure consistency and accuracy. The second operator acted as a quality-control reviewer; therefore, no formal inter-observer agreement metrics were calculated.

For each patient, a three-dimensional volume of interest (VOI) was defined to encompass the entire visible lesion while excluding adjacent non-pathological structures.

The images analyzed included conventional CT and cone beam CT (CBCT). To ensure homogeneity of the extracted radiomic data, imaging was preprocessed using isotropic resampling and gray-level discretization, in accordance with the recommendations of the Image Biomarker Standardization Initiative (IBSI) [10]. Specifically, isotropic resampling and intensity discretization were applied to standardize voxel scales and intensity distributions across different imaging protocols. In the software, a fixed bin count of 64 and a fixed bin width of 62 were used, without additional filters. This approach follows IBSI recommendations for reducing inter-scanner variability and ensuring the comparability of radiomic features across heterogeneous imaging modalities. In fact, CBCT gray values are not standardized in Hounsfield units and exhibit scanner-dependent variability; therefore, the bin width serves as a uniform resampling step rather than a physical HU measurement, allowing CT and CBCT intensity ranges to be mapped onto a comparable discretized scale. Segmentation was performed on axial, coronal, and sagittal planes, with automatic interpolation between slices to ensure volumetric continuity. VOIs were saved in a format compatible with the radiomic module of LIFEx and subsequently subjected to qualitative verification by a second operator to ensure inter-observer consistency.

For multifocal lesions, the most representative lesion, based on size and morphology, was segmented. VOIs were then used to extract morphological, first-order, and texture features [11,12], in accordance with the IBSI guidelines [10].

Subsequent analyses were conducted using Python (version 3.10) scripts developed and executed in Google Colab, a cloud-based environment that enables interactive data processing and direct integration with scientific libraries such as pandas (version 1.5.3), scikit-learn (version 1.2.2), XGBoost (version 1.7.5), and Matplotlib (version 3.7.1) [13,14,15]. The use of Colab facilitated radiomic file management, statistical analysis, data normalization, machine learning model training, and automatic generation of Excel reports. All files were manually uploaded via the user interface and saved locally at the end of each session.

### 2.2. Standardization and Normalization of Features

Radiomic data were collected in CSV/Excel format, cleaned of non-informative variables, and harmonized across cases by retaining only numerical features common to all patients. Constant or single-value variables were removed. Missing values were imputed using the median (SimpleImputer). Each case was assigned a binary diagnostic label (benign = 0 vs. malignant = 1).

To address the heterogeneous scale and distribution of radiomic features, Z-score standardization (StandardScaler) was applied after imputation. Although Min–Max normalization was computed for exploratory comparison, only Z-score standardized data were used in the final machine learning pipeline, as this approach ensures stable scaling across features and is required for the correct functioning of linear and distance-based algorithms (e.g., LASSO, SVM, ANN) while remaining fully compatible with scale-invariant tree-based models (random forest, XGBoost).

### 2.3. LASSO-Based Feature Selection and Nested Cross-Validation Framework

Within the PILOT COHORT, to develop the classification model, LASSO logistic regression with L1 regularization [16] was performed. This approach enables simultaneous classification and feature selection by shrinking less informative coefficients to zero. The optimal regularization parameter (C) was selected via 5-fold cross-validation, using grid search and AUC as the scoring metric. Model generalizability was assessed using nested cross-validation (5 outer folds × 5 inner folds). For each outer fold, a separate grid search was performed on the training set to select the best hyperparameter, and the resulting model was evaluated on the held-out test fold. This procedure prevents information leakage and provides an unbiased estimate of model performance. To assess feature stability, we recorded the non-zero coefficients selected in each outer fold. Features selected in at least three folds were considered stable. Their selection frequency and average absolute coefficient were used to rank importance. Features were then categorized into radiomic families (shape, intensity, texture, histogram) to support interpretability. No filtered images (e.g., wavelet/LoG) were used. Model performance was quantified using the area under the receiver operating characteristic curve (AUC) and a non-parametric 95% confidence interval (CI) for the AUC was computed using the percentile method, based on the empirical distribution of the five AUC values obtained from the outer folds. Specifically, the 2.5th and 97.5th percentiles were used as the lower and upper bounds of the CI.

### 2.4. Ranking of Predictors and Construction of the Final Feature Set

For each feature, we computed:

- the mean coefficient across the five outer folds;
- the mean absolute coefficient, representing its average predictive weight;
- the selection frequency, representing its stability.

Features were then ranked using a two-level hierarchy:

1. Primary criterion: frequency.
  Features appearing in more folds were considered more stable and therefore more reliable. Only features selected in at least 3 out of 5 outer folds were retained.
2. Secondary criterion: magnitude of the mean absolute coefficient.
  Among the features with equal stability, those with larger absolute coefficients were prioritized, as they contributed more strongly to the model’s decision boundary.
3. Third criterion: first/morphological and second order.
  Among the features meeting the previous criteria, the selection should, whenever possible, include—provided that at least two meaningful predictors are present for both malignant and benign lesions—at least one first-order or morphological feature and one second-order (texture) feature for each category. This ensures that the final model incorporates complementary descriptors capturing both intensity/shape information and textural heterogeneity in both benign and malignant groups.

This combined ranking allowed us to identify the most influential features overall as well as the strongest predictors of benign lesions (features with the most negative mean coefficients) and the strongest predictors of malignant lesions (features with the most positive mean coefficients).

The final number of predictors included in the model was constrained by the events-per-variable (EPV) principle [17]. To maintain statistical reliability and reduce overfitting, we adopted a conservative threshold of EPV ≥ 10–15, consistent with established recommendations for logistic regression and radiomic modeling. Based on the number of malignant cases (events) in the dataset, we calculated the maximum allowable number of predictors. The final model included the top-ranked features up to this EPV-compatible limit, ensuring methodological rigor and clinical interpretability.

### 2.5. Machine Learning Protocol

Radiomic features selected from the training cohort were used to develop and validate multiple machine learning classifiers. The binary outcome (Real Diagnosis) was already encoded numerically (1 = malignant, 0 = benign).

### 2.6. LASSO Model Training

A penalized logistic regression model with L1 regularization (LASSO) was implemented to perform simultaneous classification and feature shrinkage. The regularization parameter C = 100 was selected via prior cross-validation optimization on the full radiomic feature set.

To ensure reproducibility, all stochastic components of the LASSO model were initialized using a fixed random seed (42). The final LASSO model was trained on the complete training dataset using only the selected radiomic features, and its performance was subsequently evaluated on the independent test set.

### 2.7. Nested Cross-Validation for Model Comparison

To obtain an unbiased estimate of generalization performance for alternative classifiers, we implemented a nested 3 × 3 cross-validation framework.

The outer loop (3 folds) provided independent validation splits, while the inner loop (3 folds) performed hyperparameter tuning via grid search using AUC as the scoring metric.

The following classifiers were evaluated [18,19,20,21]:

- Random forest;
- Support vector machine (linear kernel);
- Artificial neural network (single hidden layer);
- XGBoost classifier.

Both the outer and inner cross-validation loops were executed with shuffle = True and a fixed random seed (42) to guarantee reproducibility of the fold partitions. This nested structure prevents information leakage between hyperparameter optimization and model evaluation, providing an unbiased estimate of each model’s expected performance. Given the limited dataset size and the higher computational cost of these models, a 3 × 3 configuration offered the optimal balance among statistical reliability, adequate sample size per fold, and computational feasibility. Larger nested schemes (e.g., 4 × 4 or 5 × 5) were avoided to prevent excessive computational burden and instability in the inner-loop hyperparameter tuning. A summary of the fixed and tuned hyperparameters is reported in Table 1.

### 2.8. Final Training and External Test Evaluation

After nested cross-validation, each classifier was retrained on the full training dataset using the hyperparameters identified in the inner loop as optimal.

The final models were then evaluated on the independent test set. For each classifier, we computed the predicted probabilities, class labels, ROC curves, confusion matrices, accuracy, sensitivity, specificity, and the optimal decision threshold based on Youden’s index [22,23].

### 2.9. Bootstrap Confidence Intervals

To quantify uncertainty in test-set AUC estimates, we applied a non-parametric bootstrap procedure with 5000 resamples. For each bootstrap iteration, a new dataset was generated by sampling the test set with replacement, and the AUC was recalculated. A fixed random seed (42) was used to ensure reproducibility of the bootstrap sampling process. The 95% confidence interval was defined by the 2.5th and 97.5th percentiles of the resulting bootstrap distribution, providing a robust, distribution-free estimate of variability in model performance.

**Figure 2:**
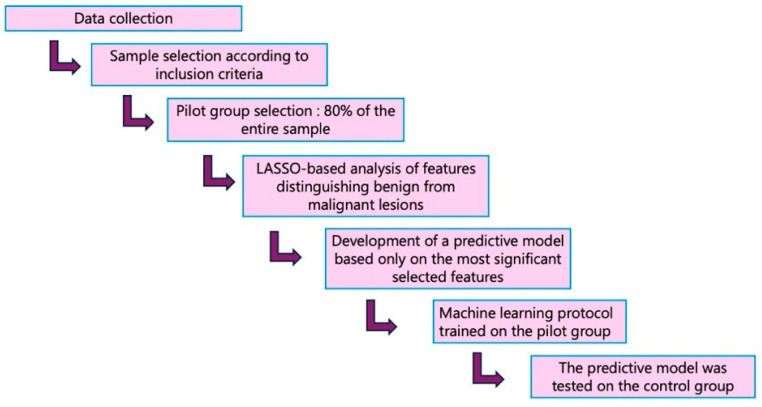
Study workflow.

The study workflow is shown in Figure 2, and a summary of the Python scripts is presented in Table 2.

### 2.10. Artificial Intelligence Assistance

Microsoft Copilot (version January 2026) was used to support the drafting and refinement of Python scripts employed for the radiomics analysis. All algorithmic design, data processing steps, and methodological decisions were performed and verified by the authors.

## 3. Results

A total of 800 histopathological examinations were analyzed from the Pathological Anatomy Service database involving patients with pathologies affecting the maxillofacial region. Out of these patients, 130 were selected; all presented with a lytic lesion of the jaws that could be classified under the previously reported diagnoses. Eighty subjects had benign conditions: 54 cysts, 15 ameloblastomas, and 11 keratocysts; 50 had malignant conditions: 6 osteosarcomas and 44 squamous cell carcinomas.

**Figure 3:**
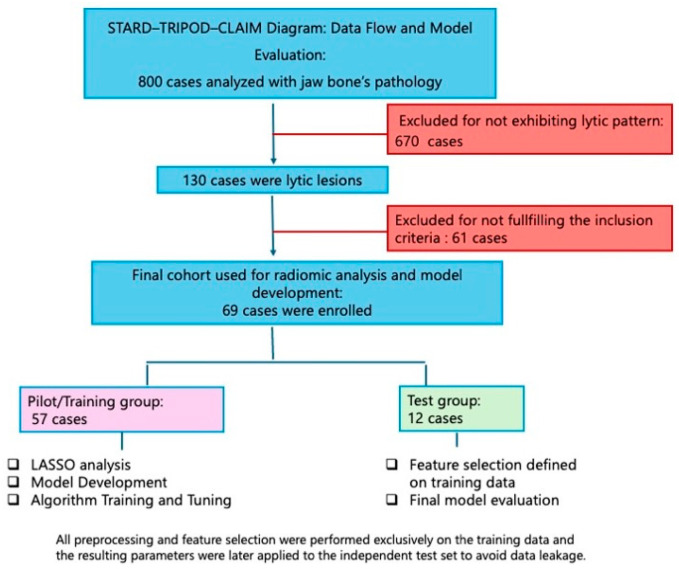
Patient flow through the study, in accordance with STARD/TRIPOD/CLAIM guidelines.

Of the 130 selected patients, 41 benign and 20 malignant lesions were excluded because the corresponding preoperative CT scans were not available in the Imaging Diagnostic Service database, as they were likely performed elsewhere. The final sample included a group we called a “pilot cohort”, consisting of 57 subjects, of which 23 had malignant lesions and 34 had benign lesions, and an independent “test cohort” consisting of 12 subjects, 5 with benign lesions, and 7 with malignant lesions. The malignant lesions in the pilot cohort were divided, based on histology: 3 patients had osteosarcoma of the jaws, and the remaining 20 had squamous cell carcinoma; the benign lesions in this sample included 6 ameloblastomas, 3 keratocysts, and 25 cysts. In the test cohort, all malignant lesions were squamous cell carcinomas, except for 1 squamocellular carcinoma arising on ameloblastoma; the benign lesions comprised 1 ameloblastoma, 1 keratocyst, and 3 cysts. The patient flow in the study is shown in the Figure 3, in accordance with STARD/TRIPOD/CLAIM guidelines.

### 3.1. Results of the LASSO-Based Feature Selection and Nested Cross-Validation Framework

Internal validation of the LASSO model identified a consistent set of radiomic features with discriminative power between benign and malignant lesions. Malignancy was coded as class 1, and benign lesions as class 0. Association direction was derived from the sign of the LASSO coefficients. Positive coefficients indicated a direct association with the malignant class, while negative coefficients indicated an inverse association, consistent with benign lesions.

The LASSO model, evaluated using a 5 × 5 nested cross-validation, demonstrated consistently high discriminative performance across all outer folds. The AUC values for the five outer validation sets were 0.77, 0.83, 0.89, 0.91, and 0.96, yielding a mean AUC of 0.87 with a standard deviation of 0.07. The 95% confidence interval, computed non-parametrically from the empirical distribution of the outer-fold AUCs, ranged from 0.78 to 0.96, indicating robust generalization despite the limited sample size.

Feature stability analysis revealed a highly reproducible radiomic signature. Many features were selected in all five outer folds (frequency = 5/5), demonstrating strong stability of the LASSO coefficients across resampling. These highly stable predictors spanned multiple radiomic families, including GLCM (Gray Level Co-occurrence Matrix), GLRLM (Gray Level Run Length Matrix), GLSZM (Gray Level Size Zone Matrix), intensity-based, and morphological features. This pattern reflects biologically plausible differences in lesion heterogeneity, attenuation, and shape between benign and malignant nodules. Additional features showed intermediate stability (frequency 3–4/5), whereas only a small subset showed inconsistent performance (1–2/5), suggesting that the nested design effectively filtered out noise-driven predictors.

To complement the stability analysis, we examined the absolute magnitudes of the LASSO coefficients to identify the features with the strongest contributions to the decision function. The features with the largest absolute coefficients overlapped substantially with those showing the highest stability, confirming that the most reproducible predictors were also those with the greatest discriminative weight. This convergence between stability and coefficient magnitude supports the robustness of the selected radiomic signature. The most significant features have been reported in Table 3. Importantly, the number of selected features was evaluated using the events-per-variable (EPV) rule. Given the sample size of 57 subjects, the maximum number of predictors that can be reliably included in a logistic model without risking overfitting is approximately six. The LASSO-based feature selection respected this constraint by shrinking most coefficients to zero and retaining only a small, stable subset of predictors across folds. This ensures that the resulting radiomic signature is not only statistically reproducible but also compliant with the established methodological standards for model parsimony.

Among the features selected in all outer folds (frequency = 5/5), five predictors showed the highest absolute LASSO coefficients and therefore contributed most strongly to the model’s discrimination. Two features—CenterOfMassShift (mean coefficient = −1.390) and IntensityBasedEnergy (mean coefficient = −1.159)—showed large negative coefficients, indicating that higher values were more frequently associated with benign lesions. This suggests that in our dataset, benign lesions tended to show greater displacement between geometric and intensity centroids and higher intensity uniformity. Conversely, three features exhibited positive coefficients and were therefore preferentially associated with malignancy: MaximumHistogramGradientGrayLevel (1.139), reflecting abrupt intensity transitions; Maximum3DDiameter (0.932), consistent with the larger size of malignant lesions; and the distance between the maximum-intensity voxel and the lesion centroid (0.894), indicating eccentric localization of dense components, a hallmark of irregular malignant growth. Together, these features capture complementary aspects of lesion morphology, intensity distribution, and textural complexity, providing a biologically plausible and statistically robust radiomic signature.

### 3.2. Results of the Ranking of Predictors and Construction of the Final Feature Set

After analyzing the radiomic features, selection was performed according to the criteria described in the previous section to identify the subset of predictors for building the model on which the machine learning protocol was applied.

Importantly, the number of selected features was evaluated using the events-per-variable (EPV) rule. Given the sample size of 57 subjects, the maximum number of predictors that can be reliably included in a logistic model without risking overfitting is approximately six. The final features selected were five, as only these met all three predefined criteria.

The model identified the following features, as shown in Table 4.

DifferenceEntropy and ShortRunLowGrayLevelEmphasis did not appear in the previous table of the most influential predictors because that table was based exclusively on selection frequency and absolute coefficient magnitude. However, for the construction of the final model, we applied an additional selection criterion: whenever at least two meaningful predictors were available for both malignant and benign lesions, the feature set should include at least one first-order or morphological descriptor and one second-order (texture) descriptor for each category. According to this rationale, DifferenceEntropy and ShortRunLowGrayLevelEmphasis were retained to ensure a balanced representation of intensity/morphological and textural information.

Biologically, DifferenceEntropy quantifies the randomness and irregularity of gray-level differences within the lesion and describes the entropy between adjacent pixels. In our model, this feature showed a negative coefficient, indicating that higher values were more frequently associated with benign lesions. Although this may appear counterintuitive, it can be explained by the fact that benign lesions, unlike malignant ones, tend to exhibit greater local heterogeneity but a higher degree of overall homogeneity, as demonstrated by the intensity-based energy feature.

Conversely, ShortRunLowGrayLevelEmphasis measures the prevalence of short homogeneous runs of low-intensity voxels, capturing fine-scale texture patterns. This feature showed a positive coefficient, meaning that higher values were preferentially associated with malignant lesions. This pattern is consistent with the presence of compact low-density structures or fine-scale architectural alterations that may reflect early necrotic foci, stromal reorganization, or micro-structural irregularities typical of malignant growth.

### 3.3. Machine Learning Protocol and External Test Evaluation

Model training revealed significant differences in predictive performance across the tested architectures. Five classification algorithms were compared: LASSO, random forest, support vector machine (SVM), artificial neural network (ANN), and XGBoost.

After training the various machine learning classifiers on the constructed model, their performance was evaluated on the unseen test data.

On the independent external test set, the LASSO classifier built on the five selected radiomic features achieved the strongest overall performance. LASSO reached an AUC of 0.83, with a bootstrap-corrected AUC of 0.83 (95% CI: 0.52–1.00). The model achieved an accuracy of 0.83, sensitivity of 0.71, and specificity of 1.00, with no false positives and two false negatives. The optimal threshold identified by Youden’s index was 0.63.

Among the alternative models, the linear SVM achieved a comparable AUC (0.83) but exhibited 0% sensitivity, classifying all malignant cases as benign. The ANN achieved an AUC of 0.77, with moderate sensitivity (0.57) and perfect specificity (1.00). Random forest and XGBoost showed substantially lower discriminative ability (AUC 0.57 and 0.37, respectively), with poor sensitivity and inconsistent probability calibration.

These results indicate that LASSO provided the most balanced and clinically meaningful performance on the external test set.

#### 3.3.1. Nested 3 × 3 Cross-Validation

Nested cross-validation confirmed the superiority of linear models:

- SVM: mean AUC = 0.77 (range 0.65–0.93);
- ANN: mean AUC = 0.62;
- Random Forest: mean AUC = 0.59;
- XGBoost: mean AUC = 0.53.

Non-linear models showed greater variability across folds and consistently lower performance, suggesting limited generalizability in this dataset.

#### 3.3.2. ROC Curves

**Figure 4:**
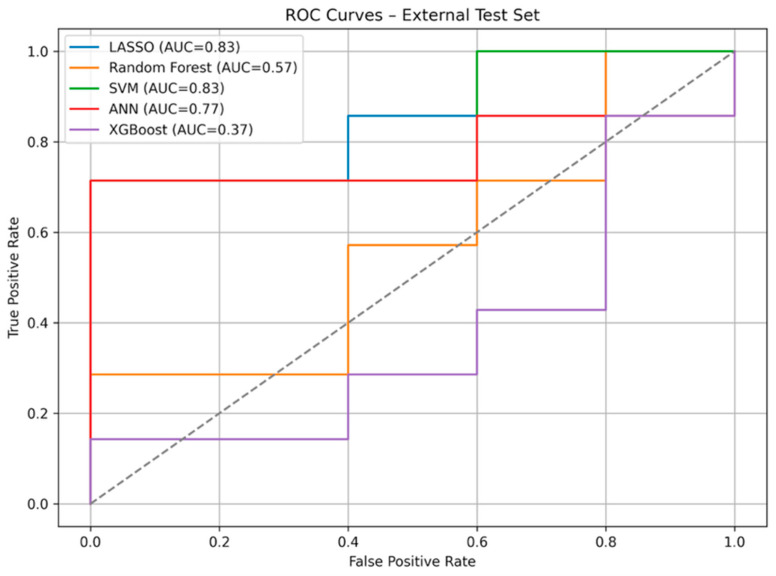
Comparison of ROC curves. Dataset: Test set with 12 subjects.

The ROC curves further highlighted the advantage of linear models. LASSO and SVM achieved the highest AUC values (0.83), although only LASSO translated this into balanced sensitivity and specificity. ANN showed intermediate performance (AUC 0.77), while random forest and XGBoost performed poorly, with curves close to the diagonal reference line. These findings reinforce the robustness of regularized linear approaches in small radiomic datasets. A comparative analysis of the ROC curves is shown in Figure 4.

#### 3.3.3. Confusion Matrices

The LASSO confusion matrix showed a balanced error profile: all benign cases were correctly classified, and only two malignant lesions were missed. This resulted in 100% specificity and 71% sensitivity, consistent with the model’s strong AUC and clinically favorable behavior.

The SVM confusion matrix revealed a critical limitation: although all benign cases were correctly identified, all malignant cases were misclassified as benign. This produced 0% sensitivity, indicating that the model’s high AUC did not translate into clinically usable performance.

The ANN correctly classified all benign cases and four malignant cases, misclassifying three malignant lesions. This yielded 57% sensitivity and 100% specificity, reflecting moderate discriminative ability but lower reliability than LASSO.

Random forest correctly identified all benign cases but misclassified five malignant lesions, achieving 29% sensitivity. This pattern suggests a tendency to under-detect malignancies and limited generalization capacity.

XGBoost showed the weakest performance, with both low sensitivity (14%) and low specificity (60%). The model misclassified most malignant cases and produced several false positives, consistent with its very low AUC.

**Figure 5:**
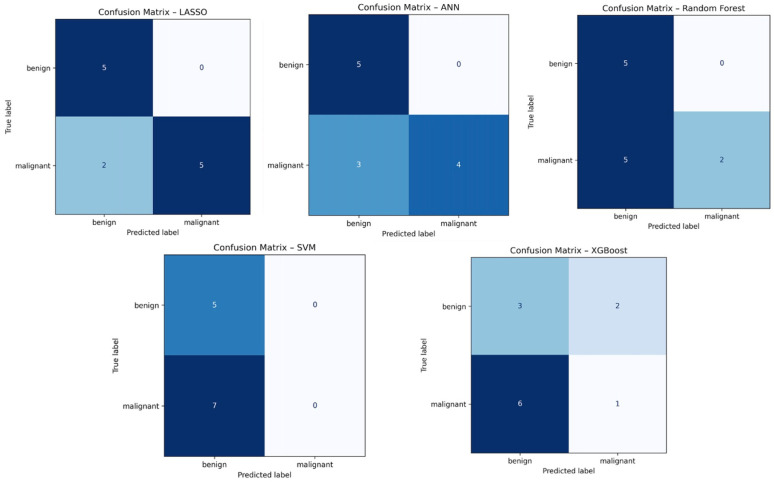
Confusion matrices of the machine learning classifiers.

Confusion matrices are reported in Figure 5.

## 4. Discussion

This study analyzed a total of 69 lytic lesions of the jaws, including both *pilot* and *test* cohorts, using radiomics applied to CBCT, dental CT (dentascan), and maxillofacial CT images, with the aim of distinguishing between benign and malignant lesions. Unlike previous studies based on panoramic radiography, this research employed CT imaging, which enables three-dimensional assessment of lesion volume, consistent with the principles of radiomics. Panoramic radiography, being a two-dimensional technique, does not allow for the extraction of complex morphologic and textural features, thereby limiting quantitative analysis.

In our study, due to the low number of ameloblastomas and keratocysts, the analysis was conducted in a binary mode: malignant lesions (carcinomas and sarcomas) versus benign lesions (cysts, keratocysts, and ameloblastomas). This choice increased the model’s statistical power and focused attention on the clinically most relevant discrimination.

In the literature, several studies have demonstrated the potential of radiomics in differentiating odontogenic lesions [24]. Muraoka et al. [25] developed a radiomic model on CT to distinguish among odontogenic cysts, keratocysts, and ameloblastomas, achieving accuracies of 0.59 and 0.60 in the training and test sets, respectively. That study focused exclusively on benign lesions. The present work differs by including malignant tumors, thereby expanding the application of radiomics in maxillofacial oncology.

Radiomics has proven particularly effective in quantifying patterns invisible to the human eye, such as texture, heterogeneity, and intensity distributions. Although the diagnostic sensitivity of radiologists is generally high, as highlighted by Giraldo-Roldán et al. [26], there are clinical scenarios in which visual interpretation may be misleading, specifically when small malignant lesions may appear minimally aggressive and fail to be recognized, or when large benign lesions may mimic malignant patterns, leading to overdiagnosis.

In these cases, radiomics offers an objective advantage by leveraging numerical metrics and predictive models that reduce inter-observer variability. However, radiomics also has limitations: very small lesions may not provide sufficient volume for meaningful feature extraction, increasing the risk of false negatives. This issue was also emphasized by Santos et al. [27], who recommended caution when applying radiomics to low-volume or poorly defined lesions.

Moreover, İçöz et al. [28] demonstrated that certain radiomic features, such as sphericity and large area emphasis, show significant differences among radicular cysts, dentigerous cysts, and keratocysts, suggesting that radiomics may serve as a non-invasive tool for differential diagnosis.

Several recent studies have confirmed the value of CBCT as a radiomic tool for characterizing benign and malignant maxillofacial lesions. Research conducted by Hung et al. [29], highlighted that volumetric CBCT images, when subjected to textural and morphological analysis, can effectively discriminate between benign and malignant lesions. Malignant lesions are characterized by greater internal heterogeneity, irregular and invasive margins, and a more heterogeneous voxel distribution, whereas benign lesions tend to exhibit sharp contours and more uniform textures.

In our study, benign lesions were characterized by greater displacement between geometric and intensity centroids and by higher intensity uniformity, reflecting their more homogeneous internal structure. In contrast, malignant lesions were associated with three features showing positive coefficients: MaximumHistogramGradientGrayLevel, indicating abrupt intensity transitions; Maximum3DDiameter, consistent with their larger size; and the distance between the maximum-intensity voxel and the lesion centroid, reflecting the eccentric localization of dense components typical of irregular malignant growth.

The features that can be directly compared with the data available in the literature are:

- **IntensityHistogramMode**, associated with benignity, reflects regular structure and uniform intensity distribution, in line with the observations of İçöz et al. [28].
- **Maximum3DDiameter**, associated with malignancy in this study, indicates irregular geometries and extended volumes and have been already validated in malignant salivary gland tumors and cervical lymph node metastases and are consistent with the expected characteristics of malignant osteolytic lesions of the jaws [29].
- At the textural level, **DifferenceEntropy**, which describes the entropy between adjacent pixels, suggests that cysts exhibit limited internal variation but less diffuse structural complexity. This aligns with the observations of Hung et al. [29], who noted that entropy and zonal distribution are among the most discriminative parameters.

In our feature selection process, we adopted a hybrid strategy that combined LASSO-based ranking with the inclusion of features from different radiomic families. Although a purely data-driven selection would have yielded slightly higher performance in our dataset, we considered it important to retain descriptors capturing complementary aspects of lesion heterogeneity. Radiomics, unlike conventional radiology, is specifically designed to quantify subtle tissue characteristics that are invisible to the human eye and that approximate microstructural and biochemical information typically assessed through histopathology. For this reason, we included at least one texture-related feature even though, due to the small sample size, texture descriptors did not emerge among the top LASSO-selected variables. This choice prioritizes biological plausibility and interpretability over maximal model performance.

After training the model, it showed the following performance on the test set. LASSO achieved the best overall performance, with an AUC of 0.83 on the external test set and perfect specificity. This behavior aligns with the expected strengths of L1-regularized models, which effectively reduce dimensionality, mitigate overfitting, and enhance generalizability when the number of features is high relative to the number of samples.

The nested cross-validation results further support this conclusion: both LASSO and SVM outperformed non-linear models, showing higher and more stable AUC values. In contrast, random forest, ANN, and XGBoost exhibited lower performance and greater variability, suggesting that these more flexible classifiers require substantially larger datasets to avoid overfitting and to fully exploit complex radiomic patterns. Although the SVM classifier achieved a relatively high AUC, it misclassified all malignant lesions as benign. This apparent discrepancy arises because AUC reflects the decision function’s ranking performance rather than its final binary predictions. In our dataset, the SVM produced a well-ordered separation of scores but an inadequate decision threshold, likely due to class imbalance and the model’s non-probabilistic nature, resulting in a sensitivity of zero despite a good AUC.

From a clinical perspective, the absence of false positives in the LASSO model is particularly relevant, as it reduces the risk of unnecessary diagnostic procedures or overtreatment. However, the presence of two false negatives indicates that the model may still miss a subset of malignant lesions. This highlights the potential benefit of integrating radiomic features with additional clinical, morphological, or molecular information to further improve sensitivity.

Overall, the findings support the use of linear penalized models as a robust, interpretable, and generalizable approach for radiomic classification in small-to-moderate datasets. Furthermore, its transparency is a crucial advantage: each feature has an interpretable coefficient, allowing for clear understanding of its contribution to classification. In clinical practice, this is fundamental for justifying diagnostic decisions and integrating the model into medical reasoning. The LASSO model allows for clear identification of the features responsible for classification, facilitating integration with clinical reasoning. Model transparency is particularly relevant in the clinical setting, where diagnostic decisions must be understood and justified. The combination of stability selection, nested cross-validation, and external testing provides a strong methodological foundation and strengthens the reliability of the proposed model. However, a direct comparison with previous radiomics studies is challenging, as no published work has investigated malignant versus benign jaw lesions using combined CT/CBCT imaging.

The consistency of confidence intervals across different phases suggests that the LASSO model maintains good predictive stability, with a limited risk of overfitting. The use of bootstrapping enabled robust estimation of performance variability, thereby reinforcing the reliability of the results.

Although calibration and decision-curve analysis are essential for assessing clinical utility, these tools were not applied in the present pilot study due to the limited sample size of the test set. Future studies with larger cohorts will be required to evaluate whether the model provides a reliable probability estimate and a measurable net clinical benefit in real-world decision-making.

Given the relatively small size of the independent test cohort, the diagnostic performance of the proposed model should be considered preliminary. Larger and more diverse datasets will be required to confirm these findings and strengthen the robustness of the predictive framework.

In relation to these findings, despite the good generalizability of the generated model, increasing the sample size is necessary to select additional features that further characterize benign and malignant patterns, thereby improving model performance. In fact, the relatively small sample size may limit the statistical power of the analysis and reduce the generalizability of the findings. The relatively small sample size of our cohort (*n* = 69), and in particular, the limited number of cases in the independent test set (*n* = 12), inevitably affects the stability and generalizability of the model. Moreover, the absence of an a priori sample size calculation represents an additional limitation, as the study was not powered to detect predefined performance thresholds. Class imbalance may have further contributed to variability in the estimates. Larger, multicenter cohorts will be necessary to confirm the robustness of the proposed model. Additionally, the imaging data were acquired using different scanners and protocols, which may introduce variability in radiomic feature extraction. Although this reflects real-world clinical conditions, it may also affect feature stability and model performance. Other limitations of the study should also be noted. Although this study primarily sought to develop a discriminative model between benign and malignant lesions—which is of greatest clinical relevance—it is necessary to further investigate multiclass classification, given the internal heterogeneity across categories. This would enable the precise diagnosis of individual lesion types. Finally, another limitation, intrinsic to radiomics itself, is the current inadequate characterization of lesions smaller than 1 cm, which, compared with biopsy, corresponds to insufficient tissue sampling. Radiomics may complement biopsy planning by identifying heterogeneous regions; however, histopathology remains the reference standard.

Radiomics-based analysis may offer a valuable adjunct to conventional radiological assessment by providing quantitative information that is not visually appreciable on standard imaging. In the context of jaw lesions, where different entities may present with overlapping morphological features, the proposed model could support clinicians by improving the early differentiation between benign and malignant or aggressive lesions. Such an approach may help reduce diagnostic uncertainty, guide decisions regarding the need for additional imaging or biopsy, and potentially streamline patient management. Although further validation is required, the integration of radiomics into routine workflows could enhance diagnostic confidence and contribute to more personalized and timely clinical decision-making.

## 5. Conclusions

Radiomics in the evaluation of cystic and lytic neoplastic lesions of the jaws currently represents a promising complementary diagnostic tool to support differential diagnosis in lesions that are difficult to classify, as well as to guide individualized treatment within a precision-medicine framework. Radiomics may support preoperative risk stratification and surgical planning but requires larger multicenter external validation before clinical deployment. In the future, with the availability of more robust datasets, radiomics may serve as a surrogate in selected cases where a biopsy could reasonably be avoided, particularly when the procedure is invasive or burdensome for the patient, such as during post-treatment reassessments after neoadjuvant or adjuvant therapies.

Although radiomics shows encouraging potential as an additional diagnostic aid, its integration into routine clinical practice will require further validation through larger, multicenter studies.

## Figures and Tables

**Table 1:** Fixed and tuned hyperparameters of the machine learning classifiers.

Model	Tuned Hyperparameters	Fixed Hyperparameters
LASSO	No nested CV in final model (C fixed)	penalty = “L1” solver = “liblinear” C = found in the model validation max_iter = 5000 median imputation standard scaling
Random Forest	n_estimators ∈ {100, 300} max_depth ∈ {None, 3, 5}	random_state = 42 median imputation
SVM	C ∈ {0.01, 0.1, 1, 10}	kernel = “linear” probability = True random_state = 42 standard scaling
ANN	alpha ∈ {0.0001, 0.001, 0.01}	hidden_layer_sizes = (10,) max_iter = 2000 random_state = 42 standard scaling
XGBoost	n_estimators ∈ {100, 300} max_depth ∈ {3, 5}	use_label_encoder = False eval_metric = “logloss” random_state = 42 median imputation

**Table 2:** Summary of Python scripts.

Python Script	Description
**Python 1—** **Internal Model Validation**	**Data Loading & Cleaning**: Import of CSV/Excel files (pandas), automatic recognition of file encoding and delimiters, removal of non-radiomic variables and duplicate entries, conversion of values to numeric format. **Data Preprocessing**: Imputation of missing values (median), normalization (Min–Max) and standardization (Z-score), construction of a cumulative dataset. **Feature Selection (LASSO)**: Logistic regression with L1 penalty, identification of variables with non-zero coefficients, selection of first order/morphological and texture features (GLCM, GLRLM, GLSZM, NGTDM).
**Python 2—** **Training Dataset Preparation**	Import of patient CSV files and normalization of identifiers to ensure correspondence with diagnostic labels. Extraction of the most significant features identified through internal validation (LASSO). Construction of a vector of selected features (≈6 variables) for each patient. Imputation of missing values using the median (SimpleImputer). Z-score standardization for statistical analysis. Min–Max normalization for visual and interpretative comparison. Saving of datasets (Z-score and Min–Max) into an Excel file with separate sheets. Integration of data into a cumulative archive to enable longitudinal analyses and subsequent updates.
**Python 3—** **Final Modelling and External Test Evaluation**	**Train/Test Split:** Import of training and independent test sets; application of previously selected radiomic features. **Final LASSO Model:** Logistic regression with C = 100 (fixed from internal validation); no further tuning; fitted on full training set and evaluated on test set. **Nested CV (3 × 3) for Other Models:** Random forest, SVM, ANN, XGBoost; hyperparameter tuning via inner CV; performance estimation via outer CV; selection of best parameters. **Final Training + Test Evaluation:** Each model fitted on the full training set with optimal parameters; performance assessed on external test set using AUC, accuracy, sensitivity, specificity, Youden index, confusion matrix. **Bootstrap Analysis:** Non-parametric bootstrap (5000 iterations) for AUC confidence intervals. **Export:** ROC curves, confusion matrices, predicted probabilities, and performance metrics saved to Excel.

**Table 3:** The most significant features highlighted in the analysis. Frequency, mean absolute coefficient (mean_coeff), and median are reported.

Feature	Frequency	Mean_Coef	Median
**MORPHOLOGICAL_CenterOfMassShift [mm]**	5	−1.390	0.103
**INTENSITY-BASED_IntensityBasedEnergy [HU]**	5	−1.159	0.025
**INTENSITYHISTOGRAM_MaximumHistogramGradientGrayLevel** **[Intensity]**	5	1.139	0.333
**MORPHOLOGICAL_Maximum3DDiameter [mm]**	5	0.932	0.248
**MORPHOLOGICAL_MaxIntensityCoor-RoiCentroidCoor-Dist [cm]**	5	0.894	10.364

**Table 4:** Selected features for the model creation. Mean absolute coefficient (Mean_Coeff) and median are reported.

Type of Lesion	Feature	Mean_Coeff	Median
**Benign**	**GLCM_DifferenceEntropy**	−0.768	0.275
	**MORPHOLOGICAL_CenterOfMassShift**	−1.390	0.103
**Malignant**	**INTENSITYHISTOGRAM_MaximumHistogramGradientGrayLevel**	1.139	0.333
	**GLRLM_ShortRunLowGrayLevelEmphasis**	−0.742	0.169
	**MORPHOLOGICAL_Maximum3DDiameter**	0.932	0.248

## Data Availability

The data presented in this study are available on request from the corresponding author due to privacy reasons.

## Abbreviations

The following abbreviations are used in this manuscript:

ANN	Artificial neural network
AUC	Area under the curve
CBCT	Cone-beam computed tomography
CI	Confidence interval
CT	Computed tomography
EPV	Events-per-variable
GLCM	Gray level co-occurrence matrix
GLRLM	Gray level run length matrix
GLSZM	Gray level size zone matrix
LASSO	Least absolute shrinkage and selection operator
ML	Machine learning
NGTDM	Neighborhood gray tone difference matrix
ROC	Receiver operating characteristic curve
SVM	Support vector machine
VOI	Volume of Interest
